# História Familiar de Hipertensão Prejudica o Balanço Autonômico, mas não a Função Endotelial em Jovens Jogadores de Futebol

**DOI:** 10.36660/abc.20180441

**Published:** 2020-07-28

**Authors:** Walter Vargas, Katya Rigatto

**Affiliations:** 1 Universidade Federal de Ciências da Saúde de Porto Alegre Porto AlegreRS Brasil Universidade Federal de Ciências da Saúde de Porto Alegre, Porto Alegre, RS - Brasil

**Keywords:** Hipertensão, Pressão Arterial, Hereditariedade/genética, Futebol, Atletas, Esportes para Jovens, Endotélio/função

## Abstract

**Fundamento:**

A história familiar de hipertensão (HFH) é um fator de risco consistente para diversas doenças crônicas que são acompanhadas por hipertensão. Além disso, a variabilidade da frequência cardíaca (VFC) e a vasodilatação mediada pelo fluxo (VMF), ambas relacionadas ao consumo máximo de oxigênio (VO2max), são geralmente prejudicadas durante a hipertensão.

**Objetivo:**

Comparar a modulação autonômica, a função endotelial (FE) e o consumo máximo de oxigênio (VO_2max_) de jovens atletas, separados de acordo com a história de pressão arterial (PA) dos seus pais, a fim de investigar a influência da ascendência genética nesses parâmetros.

**Métodos:**

Quarenta e seis jovens jogadores de futebol do sexo masculino (18±2 anos) foram divididos em quatro grupos: 1- pai e mãe normotensos (FM-N); 2- apenas pai hipertenso (F-H); 3- apenas mãe hipertensa (M-H); 4- pai e mãe hipertensos (FM-H). Foram realizadas medições da PA, VMF, VFC e do VO_2max_. Na análise estatística, foi adotado o nível de significância de 5%.

**Resultados:**

O desvio padrão dos intervalos RR normais (SDNN; FM-N=314±185; FM-H=182,4± 57,8), a raiz quadrada das médias quadráticas das diferenças dos intervalos R-R sucessivos (RMSSD; FM-N=248±134; FM-H=87±51), o número de diferenças entre intervalos NN sucessivos maiores que 50 ms (NN50; FM-N=367±83,4; FM-H=229±55), a proporção de NN50 dividida pelo número total de NNs (pNN50; FM-N=32,4±6,2; FM-H=21,1±5,3) e os componentes de alta (HF; FM-N=49±8,9; FM-H=35,3±12) e baixa frequência (LF; FM-N=50,9±8,9; FM-H=64,6±12), em unidades normalizadas (%), foram significativamente mais baixos no grupo FM-H do que no grupo FM-N (p<0,05). Por outro lado, a relação LF/HF (ms2) foi significativamente maior (p<0,05). Não foram encontradas diferenças significativas no VO2max e na VMF entre os grupos (p<0,05).

**Conclusão:**

Em jovens jogadores de futebol do sexo masculino, a HFH desempenha um papel potencialmente importante no comprometimento do balanço autonômico, principalmente quando ambos os pais são hipertensos, mas não apresentam alterações no VO_2max_ e na VMF. Nesse caso, há uma diminuição no controle simpatovagal, que parece preceder o dano endotelial. (Arq Bras Cardiol. 2020; 115(1):52-58)

## Introdução

As doenças cardiovasculares constituem a principal causa de morte no mundo.^1^ A correlação entre a pressão arterial (PA) e o risco de eventos cardiovasculares é contínuo e independe de outros fatores de risco.^2^ As últimas diretrizes para o manejo da hipertensão arterial estabelecem que os valores desejáveis de PA sistólica (PAS) e diastólica (PAD) são <120 e 80 mmHg, respectivamente.^2^ Os eventos cardiovasculares, tais como morte súbita coronária, infarto do miocárdio e acidente vascular cerebral podem facilmente ocorrer com pressões abaixo de 139/89mmHg, um limite considerado normal para a PA.^3 , 4^ Este fato indica a importância de manter a PA em valores mais baixos.

Nesse contexto, a história familiar de hipertensão surge como um importante preditor de risco a ser considerado para criar estratégias de prevenção. De fato, as diretrizes profissionais já incluem a história genética familiar na avaliação dos riscos à saúde.^5^ As evidências sugerem que a variação de 66% na PAS e 60% na PAD se deve à ascendência genética.^6^

Dados da literatura têm mostrado que os indivíduos normotensos com história familiar de hipertensão apresentam diminuição da modulação parassintética cardíaca, bem como variabilidade da frequência cardíaca (VFC). Esses achados são acompanhados por desequilíbrio simpatovagal.^7^ Além disso, tem-se postulado que esse desequilíbrio está associado ao aumento da participação simpática, que poderia ser utilizada como um marcador para o monitoramento do sistema cardiovascular.^8^ A diminuição da modulação simpática ajuda a prevenir o risco de morte prematura, até mesmo em jovens adultos não obesos,^9^ e deve ser considerada como um objetivo de tratamento de doenças do sistema cardiovascular.

Contudo, em jovens indivíduos saudáveis, há fortes indícios de que a elevação da atividade parassimpática está associada ao aumento do consumo máximo de oxigênio (VO_2max_),^10^ isto é, existe uma relação entre a modulação parassimpática e a capacidade funcional do sistema cardiovascular. Há ainda um consenso sobre uma forte relação entre o VO_2max_ e a função endotelial (FE) arterial, uma vez que eles são variáveis dependentes entre si.^11^ Entretanto, dados do nosso laboratório demonstraram que uma diferença de 10 mmHg na PA média, em grupos normotensos de jovens jogadores de futebol, é suficiente para alterar o balanço autonômico, sem alterações no VO_2max_ e na FE.^12^ Embora não seja possível concluir se a PA ou o balanço autonômico constituem uma causa ou uma consequência, esse resultado indica que a alteração no balanço autonômico provavelmente precede alterações no VO_2max_ ou na FE.

Desse modo, nosso estudo foi elaborado para comparar a modulação autonômica, a FE, e o VO_2max_ de jovens atletas, separados de acordo com a história familiar da PA dos seus pais. O objetivo foi avaliar a influência da ascendência genética naqueles parâmetros, e se os atletas normotensos apresentariam diferenças de controle do sistema cardiovascular que poderiam comprometer o seu desempenho. Adicionalmente, nosso propósito é chamar a atenção para a importância de se prevenir as doenças cardiovasculares e descobrir qual sistema é o primeiro a ser afetado nos indivíduos normotensos com uma história familiar associada à hipertensão.

## Métodos

O Comitê de Ética da Universidade Federal de Ciências da Saúde de Porto Alegre (UFCSPA) aprovou o estudo (CEP/UFCSPA número de protocolo 562.572). O tamanho da amostra foi calculado com um grau de confiança de 95%, aplicando um erro de medida tolerado estimado em 5% sobre a média do desvio-padrão variável dos intervalos RR normais (SDNN) de estudo anterior.^11^ Desse modo, para realizar este projeto de pesquisa, seria necessário um número mínimo de 39 participantes. Levando-se em conta a possibilidade de perdas e desistências em torno de 20% do número da amostra, 46 indivíduos foram convidados a participar.

Quarenta e seis jovens jogadores de futebol (18±2 anos) foram submetidos a: medições antropométricas e da pressão arterial, avaliação do sistema nervoso autonômico e da FE, testes de esforço. Todos os jogadores tinham ao menos dois anos de treinamento prévio específico de futebol e moravam nas acomodações do clube para evitar diferenças significativas no estilo de vida. Além disso, todas as refeições eram fornecidas de modo a garantir uma dieta e consumo de nutrientes semelhantes.

Antes da coleta de dados, os atletas receberam informações completas sobre os testes a serem realizados e deram consentimento informado por escrito para participar. Os dados foram coletados durante a pré-temporada de futebol, quando os atletas estavam treinando, mas não participando de uma competição. Todas as avaliações foram feitas às terças-feiras, antes do treinamento, respeitando o repouso dos atletas. Os atletas treinavam aos domingos, descansavam às segundas-feiras, e voltavam para o treinamento às terças-feiras. Para evitar enviesamento na interpretação dos dados, todas as coletas foram realizadas antes de os indivíduos serem alocados nos grupos.

Os atletas receberam instrução para comparecerem ao Laboratório de Fisioterapia/UFCSPA, às sete horas da manhã, em jejum. A PA e a FC foram medidas, seguidas de avaliação da FE na artéria braquial. Para evitar excesso de medições em um único dia, os dados antropométricos (altura, peso, idade, taxa de gordura corporal, e horário de treinamento) e o VO_2max_ foram coletados uma semana depois. Os atletas foram separados de acordo com a sua história familiar de hipertensão: 1- pai e mãe normotensos (FM-N), com 14 atletas; 2- apenas pai hipertenso (F-H), com 11 atletas; 3- apenas mãe hipertensa (M-H), com 10 atletas; e 4- pai e mãe hipertensos (FM-H), com 11 atletas. Seguindo as diretrizes para essa avaliação,^13^ a PA dos atletas foi medida, bem como a de seus pais. O *status* de hipertenso dos pais dos atletas foi definido a partir do diagnóstico realizado previamente por um médico. Desses, 53,3% estavam tomando medicação para hipertensão e 3,3% não estavam sob tratamento algum. Os indivíduos que apresentaram alterações nos valores da PA foram aconselhados a procurar atendimento médico.

### Medição da pressão arterial

Os atletas foram mantidos em ambiente calmo por pelo menos 5 minutos antes das medições da PA. Foi utilizado um método auscultatório, com os pés apoiados no chão, braço direito apoiado ao nível do coração e o manguito cobrindo pelo menos 80% da parte superior do braço. Para confirmar os dados, as medições da PA foram repetidas pelo menos duas vezes, com intervalo de 2 minutos. Quando uma diferença de mais de 6 mmHg era detectada em duas medições sucessivas, as medições eram repetidas até que a diferença fosse inferior a 4 mmHg. Para cada atleta, uma média de duas medições foi utilizada para obter a PAS.^13^

### Variabilidade da frequência cardíaca

Um monitor de frequência cardíaca (Polar modelo RS800CX, Polar Electro Oy Inc., Kempele, Finlândia) foi utilizado para coletar os dados da frequência cardíaca, (FC) com uma frequência amostral de 1000 Hz. Para a avaliação da VFC, os atletas foram orientados a permanecerem deitados em silêncio em uma maca na posição supina. Após 10 minutos, ainda na posição supina, a FC foi registrada durante 10 minutos e, em seguida, por mais 10 minutos com o atleta na posição de pé em frente à maca.^13^ O sinal foi automaticamente armazenado em intervalo RR e analisado pelo *software* Kubios HRV, versão 2.0 (University of Kuopio, Kuopio, Finland). A frequência de amostragem foi fixada a 1.000 Hz para fornecer uma resolução temporal de 1ms para cada intervalo RR, para o desvio padrão de intervalos RR normais (SDNN, ms), para a raiz média quadrática das diferenças entre intervalos RR normais sucessivos (RMSSD, ms), para o número de pares de intervalos NNs sucessivos que se diferiam em mais de 50 ms (NN50, ms), e para a proporção de NN50 dividida pelo número total de NNs (pNN50; ms).^8^

Para determinar a VFC, foi utilizado um modelo auto regressivo, com base na potência espectral integrada em duas faixas de frequência: (i) frequências altas (HF) entre 0,15 e 0,4 Hz; e (ii) frequências baixas (LF) entre 0,03 e 0,15 Hz. Os resultados foram expressos em valores absolutos (HFa and LFa, ms^2^) e seus respectivos percentuais (HFnu e LFnu, %). A relação LF/HF (ms^2^) foi calculada de acordo com a LFa e a HFa.^8^ Esta metodologia já havia sido reproduzida anteriormente em jogadores de futebol.^11^

### Avaliação da função endotelial

A função endotelial foi avaliada de modo não-invasivo, através de ultrassonografia da artéria braquial (GE, Ultrassonografia Vivid IQ, Israel) e ultrassom com Doppler, utilizando um instrumento equipado com transdutor linear de alta resolução, com frequências de 7-12-MHz (L12-3, GE Medical Systems, Israel). A ultrassonografia foi realizada em um ambiente calmo e com temperatura controlada. Em repouso, o diâmetro da artéria braquial esquerda foi medido através de imagens de ultrassom no modo B para detectar a hiperemia reativa. Antes de inflar o manguito, foi realizada uma varredura em repouso. Após a medição em repouso, o manguito foi inflado até 50 mmHg acima da pressão arterial sistólica (PAS), para ocluir o fluxo arterial por um período de cinco minutos. Este procedimento causa isquemia seguida de vasodilatação devido a mecanismos autorreguladores. Após a deflação do manguito, uma segunda varredura contínua foi registrada de 30–120 segundos. O mesmo médico ultrassonografista experiente realizou e avaliou todas as varreduras feitas por ultrassom, sem conhecer a história genética de cada atleta. Em uma posição fixa, o diâmetro do vaso foi medido “off line” com a utilização de um caliper na fase final da diástole, e coincidente com a onda R no eletrocardiograma, que estava gravando continuamente. Após um intervalo de 10 segundos, e durante o período de 30–180 segundos, a dilatação foi obtida pela diferença do valor basal. Após a liberação do manguito do esfigmomanômetro, a vasodilatação mediada pelo fluxo (VMF, %) indica o aumento no fluxo sanguíneo.^14^

### Consumo máximo de oxigênio

O Yo-Yo Intermittent Recovery Test nível 1 (Yo-Yo IRT1) foi utilizado para inferir o VO_2max_. Os atletas fizeram corridas de 2 x 20 metros, com velocidades crescentes, intercaladas com um período de recuperação ativa de 10 segundos. O teste foi controlado por sinais de áudio de um CD player e terminava quando o atleta não conseguia mais manter a velocidade para o teste. A distância percorrida naquele ponto era o resultado do teste, como descrito por Bangsbo et al.,^15^ A medição indireta do VO2max foi calculada da seguinte forma:

$${\mathrm{VO}}_{2\max}\;(\mathrm{ml}/\min/\mathrm{kg})\;=\;\mathrm{IR}1\;\mathrm{distância}\;(\mathrm{metros})\;\times \;0,0084\;+\;36,4{\;}^{14}$$

### Análise estatística

Todas as análises foram realizadas no *software* SPSS, versão 10.0 (SPSS Inc., Chicago, IL). A normalidade e a igualdade dos dados foram avaliadas pelo Teste de Shapiro-Wilk e Teste de Levene. Os resultados dos dados paramétricos foram expressos como média ± desvio padrão e os resultados dos dados não paramétricos foram descritos como mediana e intervalo interquartil.

Na análise estatística inferencial, a ANOVA de uma via foi utilizada para comparar os grupos, seguida do Teste post hoc de Tukey (quando dados paramétricos foram avaliados). O teste de Kruskal-Wallis foi utilizado para comparação entre os grupos (quando dados não paramétricos foram avaliados), e o Teste U de Mann-Whitney foi utilizado para verificar as diferenças entre os grupos. Um nível de significância de 0,05 foi adotado para todos os testes.

Para detectar uma diferença mínima de 30% entre os grupos, com uma probabilidade mínima de cometer um erro do tipo I de 5% (α = 0,05), e uma probabilidade de erro do tipo II de 20% (β = 0,2), o número mínimo de indivíduos para cada grupo foi estimado em 10, tendo como base estudo preliminar.^11^

## Resultados

Medições antropométricas, da PAS, da PAD, do consumo máximo de oxigênio e da PA dos pais

Não houve diferença significativa entre os grupos em relação à idade (17,65±0,7 anos), peso (69.25±3.6 kg), e altura (175,2±5,7 cm). Além disso, o VO_2max_ (ml/min/kg) indicou que o condicionamento físico era semelhante entre os grupos, e a PAS e a PAD (mmHg) não diferiram entre eles ( Tabela 1 ). Em relação à pressão arterial, de acordo com as definições e classificação dos níveis de pressão arterial no consultório,^3^ 15,3% dos atletas (n = 7) apresentaram PA ótima (PA<120 e 80mmHg), 39,1% (n = 18) PA normal (PA = 120-129 e/ou 80-84mmHg), e 45,6% (n = 21) PA normal alta/limítrofe (PA = 130-139 e/ou 85-89 mmHg).

**Tabela 1:** – Medições das pressões arteriais sistólica e diastólica e do consumo máximo de oxigênio

	FM-N (n=14)	F-H (n=11)	M-H (n=10)	FM-H (n=11)
**PAS (mmHg)**	124 (117-132)	128 (114-134)	128 (111-139)	128 (120-139)
**PAD (mmHg)**	72 (60-84)	76 (65-83)	79 (67-89)	78 (60-89)
**VO**_**2max**_ **(ml/kg/min)**	53,5±2,5	52,3±2,9	53,4±1,1	51,4±1,6

*PAS: pressão arterial sistólica; PAD: pressão arterial diastólica; VO_2max_.: volume máximo de oxigênio. Os valores da pressão arterial correspondem à média (intervalo de confiança) e os valores de VO_2max_ são expressos como média ± DP.*

A Tabela 2 apresenta a PA dos pais. As pressões arteriais sistólica e diastólica dos pais foram maiores no grupo de hipertensos quando comparadas com as do grupo de normotensos.

**Tabela 2:** – Medições das pressões arteriais sistólica e diastólica dos pais

	FM-N (n=14)		F-H (n=11)		M-H (n=10)		FM-H (n=11)	

	Pai	Mãe	Pai	Mãe	Pai	Mãe	Pai	Mãe
**PAS (mmHg)**	129 (120-188)	124 (120-130)	147 (130-177)	124 (120-127)	124 (120-127)	158 (143-184)	154 (130-193)	152 (130-184)
**PAS (mmHg)**	86 (75-105)	84 (77-90)	97 (85-110)	83 (77-88)	85 (80-89)	96 (80-120)	98 (85-110)	96 (80-120)

*PAS: pressão arterial sistólica; PAD: pressão arterial diastólica. Os valores correspondem à média (inrervalo de confiança).*

Medições da frequência cardíaca e da VFC no domínio do tempo e no domínio da frequência em repouso

**Figura 1:**
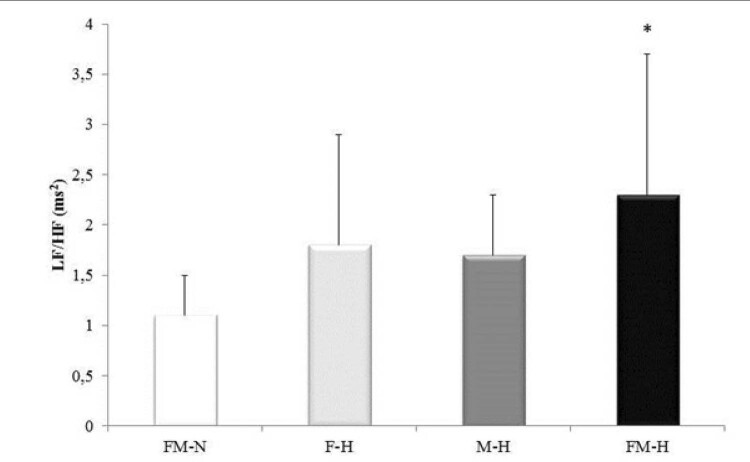
– LF/HF= Relação entre os componentes de baixa e alta frequência, isto é, o balanço autonômico dos grupos FM-N, F-H, M-H e FM-H. *Diferença entre os grupos FM-H e FM-N (p<0,005).

A VFC no domínio do tempo, observada no estudo, foi significativamente mais baixa no grupo FM-H em relação ao grupo FM-N ( Tabela 3 ). A análise espectral, pelo método no domínio da frequência (HFnu), foi significativamente mais baixa no grupo FM-H em relação ao FM-N, ao passo que os valores de LFnu e a relação LF/HF apresentaram-se significativamente maiores no grupo FM-H do que no grupo FM-N ( Figura 1 ).

**Tabela 3:** – Medições da frequência cardíaca e da VFC no domínio do tempo e no domínio da frequência em repouso

	FM-N (n=14)	F-H (n=11)	M-H (n=10)	FM-H (n=11)
**RMSSD (ms)**	210,2 (229)	179,1 (187,9)	125,2 (164,2)	82,2 (65)*
**Contagem NN50**	356±82	260±50	296±81,3	218,8±44*
**pNN50 (%)**	31,5±6,4	23,6±3,4	25,8±6,3	20,2±4,5*
**Índice triangular da VFC**	26,6±7	21,9±6,1	20,8±7,4	17,2±2,5*
**SDNN (ms)**	256 (145)	211,1 (123,1)	185,3 (84,3)	162,4 (92,7)*
**HFa (ms)**	15935 (31705,1)	13822,5 (22099,8)	3421 (24564,2)	3025,1 (15568,9)
**HFnu (%)**	48,6±8,6	40,3±13	38,4±10,3	33,8±11,2*
**LFa (ms)**	13654 (54544,1)	11575,2 (53678,3)	2591,8 (9127,9)	3173,4 (13163,2)
**LFnu (%)**	51,4±8,6	59,7±13	61,6±10,3	66,2±11,2*
**LF/HF (ms2)**	1(0,5)	1,5 (1,4)	1,8 (0,3)	2,5 (1,3)*

Os valores são apresentados como média ± DP para dados paramétricos, ou mediana (intervalo interquartil) para dados não paramétricos. *Um valor de P < 0,05 foi considerado estatisticamente significante quando comparado com o grupo FM-N. RMSSD: raiz quadrada das médias quadráticas das diferenças dos intervalos R-R sucessivos (ms); NN50: número de intervalos NNs sucessivos que diferem em mais de 50 ms; pNN50: proporção de NN 50 dividido pelo número total de NNs; VFC: variabilidade da frequência cardíaca; SDNN: desvio padrão de intervalos RR normais; HFa: componente de alta frequência absoluto; nu: unidades normalizadas; LFa: componente de baixa frequência absoluto; LF/HF: relação entre componentes de baixa e alta frequência.

### Avaliação da função endotelial

Não houve diferença significativa entre os grupos FMD ou no diâmetro basal da artéria braquial após a hiperemia reativa, antes ou depois da vasodilatação mediada por nitroglicerina ( Tabela 4 ; p>0,05).

**Tabela 4:** – Características da artéria braquial dos atletas na posição supina

	FM-N (n=14)	F-H (n=11)	M-H (n=10)	FM-H (n=11)
**B-DIA (mm)**	0,355±0,043	0,364±0,035	0,344±0,041	0,383±0,037
**RH-DIA (mm)**	0,387±0,042	0,387±0,028	0,366±0,042	0,402±0,045
**VMF (%)**	9,323±3,028	6,745±1,263	6,261±1,726	5,097±3,157
**Antes da NTG. (mm)**	0,368±0,044	0,363±0,032	0,352±0,043	0,387±0,039
**Após a NTG. (mm)**	0,431±0,039	0,431±0,036	0,419±0,041	0,453±0,034
**NTG (%)**	17,639±7,086	18,920±3,991	19,472±6,456	17,678±7,503

*B-DIA: diâmetro da artéria braquial basal; VMF: vasodilatação mediada pelo fluxo; NTG: diâmetro da artéria braquial com nitroglicerina; RH-DIA: diâmetro da artéria braquial com a hiperemia reativa. Os valores são apresentados como média ± DP.*

## Discussão

No presente estudo, não houve diferença significativa entre os grupos VMF, PAS, PAD ou VO_2max_. Dessa forma, nossos resultados sugerem que as diferenças encontradas na modulação autonômica cardiovascular entre os grupos FM-N e FM-H são decorrentes da história familiar de hipertensão dos atletas, independentemente das outras variáveis estudadas.

De acordo com dados da literatura, a prevalência de hipertensão parece atingir cerca de 30% a 45% da população geral.^13^ No nosso estudo, encontramos uma prevalência de 53,3% para os pais dos atletas ( Tabela1 ), valores acima da média mundial. Acreditamos que fatores socioeconômicos possam explicar a diferença encontrada na nossa amostra.

Nossos resultados fornecem, pela primeira vez, evidências de que a história familiar de hipertensão pode ser crucial para o desequilíbrio progressivo da regulação autonômica em jovens atletas com PA normal. Até onde sabemos, este é o primeiro estudo a mostrar um possível comprometimento precoce da modulação autonômica no processo de hipertensão. Solanki et al.,^16^ examinaram testes de função simpática em jovens não atletas do sexo masculino relacionados à obesidade, PA e hipertensão familiar. Os resultados mostraram que a função autonômica cardíaca é alterada em indivíduos com história familiar de hipertensão. Alterações no desequilíbrio autonômico em decorrência do aumento do tónus simpático foram mais acentuadas nos indivíduos com história familiar de hipertensão. Esses achados corroboram os nossos resultados, e também enfatizam a importância do exercício físico, que contrariou o desequilíbrio autonômico, dando lugar à FE normal em todos os indivíduos, independentemente do grupo experimental.^16^ Pelo menos em parte, é razoável acreditar que nossos resultados indicam que a primeira alteração no processo hipertensivo atinge os sistemas simpático e parassimpático. Essas conclusões estão em consonância com Vargas et al.,^11^ que também demonstraram que, em atletas, um pequeno aumento na PA acarreta alterações no sistema nervoso simpático, sem com isso alterar a FE ou o VO_2max_.

Levando-se em conta que a regulação autonômica pode ser avaliada por meio de uma abordagem não-invasiva para examinar a VFC nos domínios do tempo e da frequência,^8^ seria útil detectar o seu comprometimento e fornecer aos médicos dados para avaliarem a eficácia do tratamento ou, até mesmo, prevenir doenças. Apesar do enorme impacto da diminuição da VFC sobre o risco cardiovascular, não encontramos nenhuma pesquisa na literatura mostrando a correlação entre a história familiar de hipertensão e esses parâmetros em indivíduos saudáveis. Acreditamos que nossos resultados podem chamar a atenção para um método simples, de baixo custo e que pode apresentar dados associados a um risco cardiovascular significativo, como a VFC. Isso contribuirá não apenas para prevenir a hipertensão em sujeitos que estão em risco genético, mas também abrir uma nova possibilidade de monitoramento de pacientes hipertensos.

No nosso estudo, o grupo FM-H apresentou índices LFnu e relação LF/HF mais elevados quando comparado com o grupo FM-N. Ademais, no grupo FM-H, o HFnu, no domínio da frequência, e os índices SDNN, RMSSD, NN50, pNN50 e triangular da VFC, no domínio do tempo, foram significativamente menores do que aqueles observados no grupo FM-N. Esses resultados indicam que a história familiar de hipertensão é acompanhada por um aumento da modulação simpática cardíaca e por uma diminuição da modulação parassimpática, independentemente da PA normal dos jogadores de futebol.

Além disso, Tozawa et al.,^17^ buscaram determinar se a história familiar de hipertensão estaria quantitativamente associada à prevalência de hipertensão na coorte rastreada. Concluíram que o número crescente de membros da família com hipertensão tem uma correlação com um aumento na prevalência de PA mais elevada, independentemente dos fatores de risco convencionais para a hipertensão. Esses achados estão em consonância com os nossos, uma vez que também encontramos uma diferença significativa na modulação autonômica apenas quando ambos os pais eram hipertensos, o que enfatiza a importância dos antecedentes genéticos para a VFC, que é um preditor de risco cardiovascular.

Não restam dúvidas de que a atividade física está associada a efeitos benéficos para a PA. Porque o exercício é um método saudável de controle das doenças cardiovasculares,^3^ decidimos estudar apenas atletas. Nossos resultados corroboram a importância dos antecedentes genéticos. Atletas jovens e saudáveis, que tinham pai ou mãe hipertensos, apresentaram um aumento significativo da relação LF/HF, bem como uma redução na VFC.

Há também evidências consistentes demonstrando que a melhora da modulação parassimpática está associada ao aumento no VO_2max_ em indivíduos jovens saudáveis.^10^ Entretanto, no presente estudo, não foram encontradas diferenças no VO_2max_, PA e FE na comparação de todos os grupos. Isso provavelmente se deve ao fato de que, por serem compostos de jovens atletas, com dieta e consumo de nutrientes semelhantes, nossos grupos tiveram um desempenho físico elevado, o que atenuou as diferenças.

No entanto, observamos uma diferença significativa na modulação autonômica cardíaca entre os grupos FM-H e FM-N, mas não encontramos diferença significativa no VO_2max_ e na FE, o que, em última análise, manteve a PA dentro de valores normais, apesar da história familiar de hipertensão.

Em consonância com Lucini et al.,^9^ nossos resultados demonstraram que as alterações autonômicas possivelmente precedem a disfunção endotelial. Foi observado que, nos indivíduos com valores de pressão arterial enquadrados na faixa normal limítrofe, houve comprometimento da VFC. Os autores também relataram que essas alterações podem sugerir que um distúrbio na regulação autonômica anteceda o estado hipertenso,^9^ como observado na hipertensão neurogênica.

Um ponto de crítica ao nosso método é o fato de que nós não separamos os grupos de acordo com o tipo de hipertensão dos pais. Por outro lado, sabemos que a probabilidade de haver apenas pais com hipertensão neurogênica no grupo FM-H é muito baixa. Desse modo, é razoável acreditar que, apesar da causa de hipertensão, a FE foi preservada.

Conforme nossos resultados demonstraram, reforçados por estudos prévios que também buscaram respostas sobre o início do processo de hipertensão arterial,^9 , 12 , 17^ parece que a disfunção autonômica precede a disfunção endotelial. Desse modo, a descoberta de um tratamento para o desequilíbrio simpatovagal e redução o risco cardiovascular representa um desafio.

## Conclusão

Apesar de o nosso estudo apresentar limitações devido ao reduzido tamanho da amostra, ele sugere que a VFC, no domínio do tempo e da frequência, pode fornecer um desfecho funcional útil para avaliar mais precocemente o controle do sistema cardiovascular. Esse benefício se aplica a jovens saudáveis, como jovens jogadores de futebol e, acima de tudo, a pessoas sedentárias em risco. Fazer atividade física, mais do que tratar a hipertensão arterial limítrofe, representa uma alternativa para prevenir o aumento da PA por meio de estratégias que se ocupam dos mecanismos através dos quais a PA normal, no fim das contas, vem a se tornar hipertensão. Entretanto, outros estudos são necessários para confirmar essas conclusões.
