# Supplementary material for: História Familiar de Hipertensão Prejudica o Balanço Autonômico, mas não a Função Endotelial em Jovens Jogadores de Futebol
Source: Arq Bras Cardiol. 2020 Jul 28;115(1):52–8. [Article in Portuguese] doi: 10.36660/abc.20180441 (PMC8384319; doi:10.36660/abc.20180441)
Supplement: Translation

# Translation: Family History of Hypertension Impairs the Autonomic Balance, but not the Endothelial Function, in Young Soccer Players

**Authors:** Walter Vargas, Katya Rigatto

**Affiliations:** 1 Brazil Universidade Federal de Ciências da Saúde de Porto Alegre, Porto Alegre, RS - Brazil

**Keywords:** Hypertension, Blood Pressure, Heredity/genetics, Soccer, Athletes, Youth Sports, Endothelium/function

## Abstract

**Background:**

The family history of hypertension (FHH) imposes consistent risk for diverse chronic diseases that are accompanied by hypertension. Furthermore, the heart rate variability (HRV) and flow-mediated dilation (FMD) are both related to maximal oxygen uptake (VO_2max_), and are usually impaired during hypertension

**Objective:**

To compare the autonomic modulation, the endothelial function (EF) and maximum oxygen uptake (VO_2max_) of young athletes, separated according to their parents’ blood pressure (BP) history, in order to study the influence of their genetic background on those parameters.

**Methods:**

A total of 46 young male soccer players (18±2 years of age) were divided into four groups: 1-normotensive father and mother (FM-N); 2-only father was hypertensive (F-H); 3-only mother was hypertensive (M-H); 4-father and mother were hypertensive (FM-H). Measurements of BP, FMD, HRV and VO_2max_were performed. The significance level adopted in the statistical analysis was 5%.

**Results:**

The standard deviation of normal RR intervals (SDNN; FM-N=314±185; FM-H=182.4± 57.8), the square root of the mean squared differences in successive RR intervals (RMSSD; FM-N=248±134; FM-H=87±51), the number of interval differences of successive NN intervals greater than 50ms (NN50; FM-N=367±83.4; FM-H=229±55), the ratio derived by dividing NN50 by the total number of NN intervals (pNN50; FM-N=32.4±6.2; FM-H=21.1±5.3) and the high (HF; FM-N=49±8.9; FM-H=35.3±12) and low-frequency (LF; FM-N=50.9±8.9; FM-H=64.6±12) components, in normalized units (%), were significantly lower in the FM-H group than in the FM-N group (p<0.05). On the other hand, the LF/HF ratio (ms^2^) was significantly higher (p<0.05). We found no significant difference between the groups in VO_2max_and FMD (p<0.05).

**Conclusions:**

In young male soccer players, the FHH plays a potentially role in autonomic balance impairment, especially when both parents are hypertensive, but present no changes in VO_2max_and FMD. In this case, there is a decrease in the sympathetic-vagal control, which seems to precede the endothelial damage (Arq Bras Cardiol. 2020; 115(1):52-58)

## Introduction

Cardiovascular disease is the leading cause of death worldwide.^1^ The correlation between blood pressure (BP) and the risk of cardiovascular events is continuous and independent of other risk factors^2^ . The latest Guidelines for the management of arterial hypertension established that the optimal values of systolic (SBP) and diastolic BP (DBP) are <120 and 80 mmHg, respectively.^2^ Cardiovascular events, such as sudden coronary death, myocardial infarction and stroke might easily occur at pressure even below 139/89mmHg, a threshold considered as normal BP.^3 , 4^ This fact indicates the importance of keeping the BP at lower values.

In this context, family history of hypertension emerges as an important predictor of risk to be considered in creating prevention strategies. In fact, professional guidelines for health risk assessment already include the genetic family history.^5^ Evidence suggests that the variation of 66% in SBP and 60% in DPB are due to genetic background.^6^

Data from the literature have shown that normotensive subjects with a family history of hypertension have lower cardiac parasympathetic modulation and also heart rate variability (HRV). These findings are accompanied by sympathovagal imbalance.^7^ Moreover, it has been postulated that this imbalance is associated to increased sympathetic participation, which could be used as a marker for monitoring the cardiovascular system.^8^ A decrease in sympathetic modulation helps preventing the risk of premature death, even in non-obese young adults,^9^ and should be a goal for treating cardiovascular system diseases.

Nevertheless, in healthy young subjects, there is consistent evidence that enhanced parasympathetic activity is associated with an increase in maximal oxygen uptake (VO_2max_),^10^ i.e., there is a relationship between the parasympathetic modulation and the functional capacity of the cardiovascular system. There is also a consensus that there is a strong relationship between VO_2max_and arterial endothelial function (EF), since they are dependent variables.^11^ However, data from our laboratory have shown, in a normotensive group of young soccer players, that a difference of 10 mmHg in mean BP is enough to change the autonomic balance, without changing VO_2max_and EF.^12^ Although it is not possible to conclude whether BP or autonomic balance is the cause or the consequence, this result indicates that the alteration in autonomic balance probably precedes the VO_2max_or EF changes.

Thus, our study was designed to compare the autonomic modulation, the EF, and the VO_2max_of young athletes grouped according to the parents’ BP history. The objective was to access the influence of the genetic background in those parameters, and whether normotensive athletes would present differences in the cardiovascular system control that could compromise their performance. Additionally, our intention is also to drive attention for the importance of preventing cardiovascular diseases and finding out which system is the first to be compromised in normotensive subjects with a family history of hypertension.

## Methods

The Ethics Committee of the Universidade Federal de Ciências da Saúde de Porto Alegre (UFCSPA) approved the study (CEP/UFCSPA protocol number 562.572). The sample size was calculated with a confidence level of 95%, applying a tolerated measurement error estimated at 5% over the mean of variable standard deviation of normal RR intervals (SDNN) of anterior study.^11^ Thus, in order to conduct this research project, a minimum number of 39 participants was required. Predicting losses and dropouts around 20% of the sample number, 46 individuals were invited to participate.

Forty-six young male soccer players (18±2 years of age) were submitted to: anthropometric and BP measurements, autonomic nervous system and EF evaluation, and exercise tests. All players had at least two years of previous soccer-specific training and lived in the club accommodations to avoid significant differences in lifestyle. Moreover, all meals were provided assuring similar diet and nutrients intake.

Before data collection, the athletes were fully informed about the tests to be performed and provided a written informed consent to participate. The data was collected during the soccer preseason, when the athletes were training, but not participating in any competitions. All evaluations were made on Tuesdays, before training, respecting the athletes’ rest breaks. The athletes trained on Sundays, rested on Mondays, and returned to training on Tuesdays. To avoid any tendency in data interpretation, all data collection were performed before the subjects were allocated in the groups.

The athletes were instructed to attend the Laboratory of Physiotherapy/UFCSPA, at 7 a.m., fasting. The BP and HR were measured, followed by an evaluation of EF in the brachial artery. To avoid an excess of measurements in a single day, the anthropometric data (height, weight, age, body fat percentage, and time of training) and VO_2max_were collected one week later. The athletes were grouped according to their family history of hypertension: 1- normotensive father and mother (FM-N), with 14 athletes; 2- only father was hypertensive (F-H), with 11 athletes; 3- only mother was hypertensive (M-H), with 10 athletes; and 4- father and mother were hypertensive (FM-H), with 11 athletes. Following the guidelines for this assessment,^13^ the BP of the athletes was measured, as well as their parents’. The hypertensive status of the athletes’ parents was previously defined by a physician (53.3% of those individuals were taking anti-hypertensive drugs and 3.3% were not treating their state). Individuals who showed changes in BP values were advised to seek medical attention.

### Blood pressure measurement

An auscultatory method was used. The athletes were kept in a quiet environment for at least five minutes before BP measurements, seated with their feet on the floor, right arm supported at heart level and BP cuff covering at least 80% of the upper arm. To confirm the data, the BP measurement was repeated at least twice at 2-minute interval. When a difference of more than 6 mmHg was detected in two successive measurements, the measurements were repeated until the difference was less than 4 mmHg. For each athlete, an average of two measurements was used to obtain the SBP.^13^

### Heart rate variability

A Polar model RS800CX heart-rate monitor (Polar Electro Öy, Kempele, Finland) was used to collect heart rate (HR) data at a sampling frequency of 1000Hz. For the evaluation of HRV, the athletes were instructed to lie quietly on a stretcher in the supine position. After 10 minutes, still in the supine position, the HR signal was recorded for 10 minutes followed by additional 10 minutes with the athlete standing in front of the stretcher.^13^ The signal was automatically stored as an RR interval and analyzed with Kubios HRV software version 2.0 (University of Kuopio, Kuopio, Finland). A 1,000-Hz sampling rate was chosen to provide a temporal resolution of 1 ms for each RR interval, a standard deviation of normal RR intervals (SDNN, ms), the square root of the mean squared differences among consecutive RR intervals (RMSSD, ms), the number of interval differences of successive NN intervals greater than 50 ms (NN50, ms), and the proportion derived by dividing NN50 by the total number of NN intervals (pNN50; ms)^8^ .

An autoregressive method was used to determine HRV, based on the spectral power integrated in two frequency bands: (i) a high-frequency (HF) band from 0.15 to 0.4 Hz; and (ii) a low-frequency (LF) band from 0.03 to 0.15 Hz. The results were expressed in absolute values (HFa and LFa, ms^2^) and their respective percentages (HFnu and LFnu, %). The LF/HF ratio (ms^2^) was calculated according to the LFa and HFa.^8^ This methodology had been previously reproduced in the soccer players.^11^

### Endothelial Function Assessment

EF was assessed noninvasively by means of a brachial artery ultrasound probe (GE Medical Systems, Vivid I Ultrasound, Israel) and Doppler ultrasonography, using an instrument equipped with a 7- 12-MHz high-resolution linear probe (L12-3, GE Medical Systems, Israel). The ultrasonography was performed in a silent, temperature-controlled room. At rest, the left brachial artery diameter was measured by B-mode ultrasound images to detect reactive hyperemia. Before BP cuff inflation, a resting scan was performed. After the resting measurement, the cuff was inflated for 5 min at 50 mmHg above SBP, to occlude the arterial flow. This procedure causes ischemia followed by vasodilation due to auto-regulatory mechanisms. After the cuff deflation, a second continuous scan was recorded from 30–120 seconds. The same experienced sonographer performed and analyzed all ultrasound scans without knowing the genetic history of each athlete. At a fixed position, the vessel diameter was measured offline with ultrasonic calipers at end-diastole, and coincident with the R wave on an electrocardiogram, which was continuously recording. After an interval of 10 seconds and during the period within 30–180 seconds, the dilatation was obtained by the difference from baseline. After the release of the sphygmomanometer cuff, the flow-mediated dilation (FMD, %) indicates the increase in blood flow.^14^

### Maximal oxygen uptake

The Yo-Yo intermittent recovery test level 1 (Yo-Yo IR1) was used to infer the VO_2max_. The athletes performed 2×20-minute shuttle runs at increasing speeds, interspersed with a 10-second period of active recovery. The test was controlled by audio signals from a compact-disc player and ended when the athlete was unable to maintain the speed for the test. The distance traveled at that point was the result of the test, as described by Bangsbo et al.^15^ The indirect measurement of VO2max was calculated as follows:

$${\mathrm{VO}}_{2\max}\;(\mathrm{ml}/\min/\mathrm{kg})\;=\;\mathrm{IR}1\;\mathrm{distance}\;(\mathrm{meters})\;\times \;0.0084\;+\;36.4{\;}^{14}$$

### Statistical analysis

All analyses were performed with the SPSS software version 10.0 (SPSS Inc., Chicago, IL). The data normality and equality were assessed through the Shapiro–Wilk and Levene’s tests. The results of parametric data are presented as mean ± standard deviation, and the results of non-parametric data are described as median and interquartile range.

In the inferential statistical analysis, one-way ANOVA was used to compare the groups, followed by Tukey’s post hoc test, when parametric data. The Kruskal-Wallis test was used to compare the groups when non-parametric data, and U of the Mann-Whitney test was used to verify the differences between the groups. A significance level of 0.05 was adopted for all the tests.

To detect a minimum 30% difference between the groups with a minimum probability of a type I error of 5% (α = 0.05) and a probability of type II error of 20% (β = 0.2), the minimum number of individuals for each group was estimated at 10, based on a preliminary study.^11^

## Results

Anthropometric, SBP, DBP, maximal oxygen uptake measurements and parents’ BP

There was no significant difference among groups regarding the age (years; 17.65±0.7), weight (kg; 69.25±3.6), and height (cm; 175.2±5.7). Moreover, VO_2max_(ml/min/kg) indicated that physical fitness was similar among groups, and SBP and DBP (mmHg) were not different among the groups either ( Table 1 ). According to the definitions and classification of office blood pressure levels,^3^ the blood pressure level in 15.3% (n = 7) of the athletes was optimal BP (BP<120 and 80mmHg), 39.1% (n = 18) of them presented normal BP (BP = 120-129 and/or 80-84mmHg), and 45.6% (n = 21) had high normal blood pressure (BP = 130-139 and/or 85-89 mmHg).

**Table 1:** – Measurements of systolic and diastolic blood pressure and maximal oxygen uptake

	FM-N (n=14)	F-H (n=11)	M-H (n=10)	FM-H (n=11)
SBP (mmHg)	124 (117-132)	128 (114-134)	128 (111-139)	128 (120-139)
DBP (mmHg)	72 (60-84)	76 (65-83)	79 (67-89)	78 (60-89)
VO2max (ml/kg/min)	53.5±2.5	52.3±2.9	53.4±1.1	51.4±1.6

*SBP: systolic blood pressure; DBP: diastolic blood pressure; VO_2max_: maximal oxygen uptake. Blood pressure values are expressed as mean (confidence interval) and VO_2max_values, as mean ± SD.*

SBP: systolic blood pressure; DBP: diastolic blood pressure; VO_2max_: maximal oxygen uptake. Blood pressure values are expressed as mean (confidence interval) and VO_2max_values, as mean ± SD.

The parents’ BP are shown in Table 2 . The parents’ systolic and diastolic blood pressures were higher in the hypertensive group when compared to the normotensive one.

**Table 2:** – Measurements of parents’ systolic and diastolic blood pressure

	FM-N (n=14)		F-H (n=11)		M-H (n=10)		FM-H (n=11)	

	Father	Mother	Father	Mother	Father	Mother	Father	Mother
**SBP (mmHg)**	129 (120-188)	124 (120-130)	147 (130-177)	124 (120-127)	124 (120-127)	158 (143-184)	154 (130-193)	152 (130-184)
**DBP (mmHg)**	86 (75-105)	84 (77-90)	97 (85-110)	83 (77-88)	85 (80-89)	96 (80-120)	98 (85-110)	96 (80-120)

*PAS: pressão arterial sistólica; PAD: pressão arterial diastólica. Os valores correspondem à média (inrervalo de confiança).*

Heart rate and time-domain and frequency-domain measurements of resting heart-rate variability

In our study, the HRV in the time domain was significantly lower in the FM-H than in the FM-N group ( Table 3 ). The spectral analysis, using a frequency-domain method (HFnu) was significantly lower in the FM-H than in the FM-N group, and LFnu and the LF/HF ratio were significantly higher in the FM-H than in the FM-N group ( Figure 1 ).

**Table 3:** – Heart rate, time-domain and frequency-domain measurements of resting heart-rate variability

	FM-N (n=14)	F-H (n=11)	M-H (n=10)	FM-H (n=11)
**RMSSD (ms)**	210.2 (229)	179.1 (187,9)	125.2 (164.2)	82.2 (65)*
**NN50(count)**	356±82	260±50	296±81.3	218.8±44*
**pNN50 (%)**	31.5±6.4	23.6±3.4	25.8±6.3	20.2±4.5*
**HRV triangular index**	26.6±7	21.9±6.1	20.8±7.4	17.2±2.5*
**SDNN (ms)**	256 (145)	211.1 (123.1)	185.3 (84.3)	162.4 (92.7)*
**HFa (ms)**	15935 (31705.1)	13822.5 (22099.8)	3421 (24564.2)	3025.1 (15568.9)
**HFnu (%)**	48.6±8.6	40.3±13	38.4±10.3	33.8±11.2*
**LFa (ms)**	13654 (54544.1)	11575.2 (53678.3)	2591.8 (9127.9)	3173.4 (13163.2)
**LFnu (%)**	51.4±8.6	59.7±13	61.6±10.3	66.2±11.2*
**LF/HF (ms2)**	1(0.5)	1.5 (1.4)	1.8 (0.3)	2.5 (1.3)*

Values are expressed as mean ± SD when parametric data, or median (interquartile range) when non-parametric data. *A value of p < 0.05 was considered statistically significant when compared to the group FM-N. RMSSD: square root of the mean squared differences among consecutive RR intervals; NN50: the number of successive NN intervals greater than 50ms; pNN50; the ratio derived by dividing NN50 by the total number of NN intervals; HRV: heart rate variability; SDNN: standard deviation of normal RR intervals; HFa: absolute values of high-frequency components; nu: normalized units; LFa: absolute values of low-frequency components; LF/HF: ratio between low- and high-frequency power components.

**Figure 1:** – LF/HF= Ratio between low and high frequency power components, i.e., the autonomic balance of the FM-N, F-H, M-H and FM-H groups. *Differences between FM-H and FM-N groups (p<0.005).

### Endothelial function assessment

There was no significant difference between the groups regarding FMD or baseline brachial artery diameter upon reactive hyperemia, either before or after nitroglycerin-mediated vasodilatation ( Table 4 ; P>0.05).

**Table 4:** – Brachial artery characteristics of athletes in supine position

	FM-N (n=14)	F-H (n=11)	M-H (n=10)	FM-H (n=11)
**B-DIA (mm)**	0.355±0.043	0.364±0.035	0.344±0.041	0.383±0.037
**RH-DIA (mm)**	0.387±0.042	0.387±0.028	0.366±0.042	0.402±0.045
**FMD (%)**	9.323±3.028	6.745±1.263	6.261±1.726	5.097±3.157
**Before NTG (mm)**	0.368±0.044	0.363±0.032	0.352±0.043	0.387±0.039
**After NTG. (mm)**	0.431±0.039	0.431±0.036	0.419±0.041	0.453±0.034
**NTG (%)**	17.639±7.086	18.920±3.991	19.472±6.456	17.678±7.503

*B-DIA: basal brachial artery diameter; FMD: flow-mediated dilation; NTG: brachial artery diameter with nitroglycerin; RH-DIA: brachial artery diameter with reactive hyperemia. Values are expressed as mean ± SD.*

## Discussion

In the present study, there was no significant difference between the groups in terms of FMD, SBP, DBP or VO_2max_. Thus, our results suggest that the differences found between the FM-N and FM-H groups in relation to cardiovascular autonomic modulation are due to the family history of hypertension of the athletes, regardless of the other variables studied.

According to the literature data, the prevalence of hypertension appears to affect about 30% to 45% of the general population.^13^ In our study, we found a prevalence of 53.3% for the athletes’ parents ( Table 1 ), values above the world average. We believe that socioeconomic factors can explain the differences found in our sampling.

Our results provide, for the first time, evidence that family history of hypertension might be crucial to the progressive imbalance of autonomic regulation in healthy young athletes with normal BP. Based on our knowledge, this is the first study to show the possible early involvement of the autonomic modulation in the hypertensive process. Solanki et al.^16^ examined sympathetic function tests of young nonathletic males, considering measures of obesity, PA and familial hypertension. Their results showed that the cardiac autonomic function is altered in individuals with a family history of hypertension. Autonomic imbalance changes due to increased sympathetic tone was more pronounced in subjects with a family history of hypertension. These findings by Solanki and co-workers^16^ are in agreement with our results, and also highlight the importance of physical exercise, which countered the autonomic imbalance in favor of normal EF for all study subjects, regardless of the experimental group. At least partially, it is reasonable to believe that our results point out towards the fact that the first changes in the hypertensive process affect the sympathetic and parasympathetic systems. These conclusions are in agreement with Vargas et al.,^11^ who also showed that in athletes a small increase in BP induces changes in the autonomic nervous system without changing the EF or VO_2max_.

Considering that the autonomic regulation can be assessed with a non-invasive approach to evaluate the HRV in the time and frequency domains,^8^ it could be useful to detect its impairment and provide the physicians with valuable information to assess the treatment efficacy or even to prevent diseases. Despite the enormous impact of a decrease in HRV over the cardiovascular risk, we did not find any researches in the literature showing a correlation between the family history of hypertension and these parameters in healthy subjects. We believe that our results may drive the attention to a method that is easy, of low cost and able to present data associated to a significant cardiovascular risk, such as HRV. They will contribute not only to prevent hypertension in subjects who are at genetic risk, but also to open up a new possibility of monitoring hypertensive patients.

In our study, the FM-H group showed higher LFnu and LF/HF ratio than the FM-N group. In addition, in the FM-H group, the HFnu, in the frequency-domain, and SDNN, RMSSD, NN50, pNN50 and HRV triangular index, in the time-domain, were significantly lower than those from the FM-N group. These results indicate that family history of hypertension is accompanied by an increase in cardiac sympathetic modulation and a decrease in parasympathetic modulation, regardless of the normal BP of each soccer player.

Moreover, Tozawa et al.^17^ sought to determine whether the family history of hypertension was quantitatively associated with the prevalence of hypertension in a screened cohort. They concluded that the increasing number of family members with hypertension had a correlation with increased prevalence of higher BP, regardless of the conventional risk factors for hypertension. These findings are in agreement with ours, since we also found a significant difference in autonomic modulation only when both parents were hypertensive, emphasizing the importance of the genetic background for HRV, which is a predictor of cardiovascular risk.

There is no doubt that physical exercise is associated with beneficial effects on BP. Because exercising is a healthy method of cardiovascular diseases control,^3^ we have chosen to study only athletes. Our results emphasize the importance of the genetic background. Healthy and young athletes, who had hypertensive father and mother, presented a significant increase in the LF/HF ratio, as well as a reduction in HRV.

There is also consistent evidence showing that enhanced parasympathetic modulation is associated with an increase in VO2max in healthy young subjects.^10^ However, in the present study we have found no differences in VO_2max_, BP and EF between all groups. This is probably due to the fact that, being composed of young athletes who had similar diets and nutrients intake, our groups had a high physical performance, which attenuated the differences.

Although we have found a significant difference in cardiac autonomic modulation between the FM-H and FM-N groups, we have not found significant differences in VO_2max_and EF which, ultimately, kept BP in normal values despite the family history of hypertension.

In agreement with Lucini et al.,^9^ our results demonstrated that the autonomic changes possibly precede endothelial dysfunction. They showed that, in subjects with BP in the upper normotensive range, the HRV was impaired. These authors also reported that these changes might suggest that the disturbance in the autonomic regulation precedes the hypertensive state,9 as seen in neurogenic hypertension.

A point of criticism to our method is the fact that we did not separate the groups according to the parents’ type of hypertension. On the other hand, we know that the probability to have only parents with neurogenic hypertension in the FM-H group is very low. Thus, it is reasonable to believe that, regardless of the cause of hypertension, the EF was preserved.

According to our results, strengthened by previous studies that also looked for answers concerning the beginning of the arterial hypertensive process,^9 , 12 , 17^ it appears that the autonomic dysfunction precedes the endothelial dysfunction. Thus, it is a challenge to discover a treatment for sympathovagal imbalance and reduce cardiovascular risk.

## Conclusion

Although our study has the limitation of a small sample size, it suggests that HRV, in the time and frequency domains, may provide a useful functional outcome to assess the cardiovascular system control earlier. This advantage is useful for healthy young people, as young soccer players, and is probably more important for sedentary people at risk. Doing exercise, more than treating borderline hypertension, represents an alternative to prevent the increase in BP through strategies that treat the mechanisms by which normal BP, eventually, becomes hypertension. However, further studies are needed to confirm these conclusions.
